# Parental waivers to enable adolescent participation in certain forms of health research: lessons from a South African case study

**DOI:** 10.1186/s12910-022-00833-5

**Published:** 2022-09-24

**Authors:** Ann Strode, Zaynab Essack

**Affiliations:** 1 School of Law, University of KwaZulu-Natal, Pietermaritzburg, South Africa; 2 South African Research Ethics Training Initiative (SARETI), University of KwaZulu-Natal, Pietermaritzburg, South Africa; 3 Human and Social Capabilities Division, Centre for Community-Based Research, Human Sciences Research Council, Pietermaritzburg, South Africa

**Keywords:** Parental consent, Waivers, Adolescent research, CIOMS, South Africa, Ethics guidelines

## Abstract

**Background:**

The South African legal framework requires mandatory parental/legal guardian consent for *all* research with children. Ethics guidelines provide some reprieve by allowing RECs to grant waivers of parental or guardianship consent in certain defined circumstances. In the first instance, consent may be provided by a proxy when parents or guardians are unavailable, for example with orphaned children. In the second instance, guidelines permit adolescent self-consent when the nature of the study justifies this approach, for example, research on sensitive issues like sexual behaviour or substance use.

**Discussion:**

South African guidelines set several conditions that must be met for waivers to be granted. These norms overlap with those in international guidelines. However, the ethical norms, especially related to self-consent are sometimes vague. This article critically evaluates the consent norms in the national ethics guidelines and makes recommendations for reform to ethics guidelines in a way that recognises the value of child participation in research, their evolving decision-making capacity and their best interests.

**Conclusion:**

Recommendations are made to harmonise ethics guidelines and law in a way that promotes child participation in research, to ensure additional protections for adolescents when self-consent is allowed, and to withdraw procedural requirements for the community endorsement of self-consent strategies.

## Introduction

Adolescent research in South Africa is complex. The legal framework dealing with consent to child research is highly restrictive [1–4]. The National Health Act [5] requires mandatory parental/legal guardian consent for all forms of health research involving persons under 18 years old [1–4]. No exceptions or waivers are permitted [3]. However, the national ethics guidelines [6] offer some flexibility in consent approaches, including providing Research Ethics Committees (RECs) the authority to grant waivers of parental or guardianship consent in certain circumstances [6].

This disjuncture between law and ethics has led to some researchers and RECs bypassing the law and relying on the norms in ethics guidelines which allow adolescent self-consent through a parental waiver—justifying this approach by arguing that an internal conflict exists within the National Health Act [5]. Section 71 of the Act requires mandatory parental consent [1–5] but Sect. 73 empowers RECs to grant ethical approval where research ‘meets the ethical standards’ of the REC. Further, as ethics guidelines allow parental waivers in certain circumstances it is not unlawful to follow this ethical approach [3]. Using their authority in Sect. 73 to approve ethical research some RECs approve protocols which meet the ethical norms regarding adolescent self-consent [3]. However, this ambiguity regarding whether RECs must apply the law or ethics guidelines has led to confusion and inconsistency in approaches between RECs [3] and restricted the nature and volume of possible child health research [1–4, 7–14].

The lack of legal flexibility regarding parental waivers has a disparate impact on ‘sensitive research’ with adolescents. Such research includes studies on HIV risk behaviours, under-age sex, access to sexual and reproductive healthcare services (e.g., contraceptives, termination of pregnancy), sex work practices and research with adolescents who are sexual and gender minorities or who use drugs and alcohol. Online research with children and adolescents is also often complicated by the need to gain parental consent, even for less sensitive research such as the time children spend on cell phones daily [3]. Although this is a significant issue facing the South African ethical-legal framework, there is limited literature on parental waivers. Where it exists, it largely focuses the restrictive nature of mandatory parental consent norms in the law [1–7, 11, 12], the impact of mandatory parental consent on health research [2–4, 8, 9, 11, 12, 15], and justifications for including adolescents in research without parental consent [2, 7, 8, 11–14]. The absence of scholarship on adolescent self-consent in South Africa is a critical gap. Although the flexibility offered by ethics guidelines has enabled ethically important research [3], the guidelines are to be updated soon (personal communication, Dr Theresa Burgess, 4 April 2021) and discussion and debate on their content could inform reform.

This article examines international norms on parental waivers as set out in the Council for International Organisations of Medical Sciences (CIOMS) guidelines [16]. It then critically evaluates the South African consent norms in the national ethics guidelines [6], and explores the extent to which they are aligned to international norms. The article makes specific recommendations for guideline reform in South Africa and identifies what other jurisdictions could learn from this South African case study.

## Parental waivers in international ethics guidelines

The 2016 version of the International Ethical Guidelines for Health-related Research involving Humans, issued by CIOMS, provides a broad set of international ethical norms to guide health researchers and RECs at a country level. The guidelines recognise that while research with children is essential, children require special protections [16]. Guideline 17 requires parental permission and child assent for participation in health research [16]. In cases where children do not have parents, the permission of a legal guardian or another legally authorised person should be obtained [16]. These guidelines provide that parental waivers are ethically justified when [16]:

1. Special protections are put in place;
2. The research would not be ‘feasible’ if parental consent was mandatory or because the nature of the study makes parental consent ‘undesirable’; and
3. The best interests of the child standard is used to assess whether the waiver is appropriate.

## The ethical framework for child and adolescent consent to health research in South Africa

South Africa’s national ethics guidelines require written (in most cases) parental or guardianship consent for health research [6]. This is to be accompanied by written assent from the child [6]. The guidelines allow waivers of parental consent in two instances. In the first instance, if parents and guardians are not available to consent because the research is with orphans and vulnerable children (OVCs), proxy consent may be provided by a caregiver [6] (as defined in Sect. 1 of the Children’s Act [17]). In this instance [17]:

(i) A justification must be placed before the REC showing that the study involves ‘important research that seeks to understand and improve psychosocial, economic and educational conditions for OVCs as well as better their future wellbeing’;
(ii) It must be shown that the study cannot be done with adult participants; and
(iii) It must be demonstrated that (generally) the study meets the risk standards set out in the guidelines, that is, the study should not be more than minimal risk. If it is more than minimal risk but there is the possibility of a direct benefit, then any increase in risk should be justified by the benefit. If the study poses more than minimal risk and offers no direct benefit, the possibility of generalisable knowledge must be weighed up against the potential risks.

In the case of OVCs, the guidelines are simply allowing a broader category of adults, namely caregivers, to provide proxy consent. This is significant as it recognises the lived reality of South African children, many of whom stay with caregivers rather than parents or legal guardians [18]. In 2017, there were 2.8 million orphans in South Africa [19]. In the same period, approximately 58,000 children were living in child-headed households (CHHs) without adult supervision [19]. These parental waivers allow a highly vulnerable population to participate and benefit from health research. Usefully, the guidelines allow an increase in the risk levels to a minor increase over minimal risk [6]. They also refer to the legal definition of caregivers in the Children’s Act [17] which facilitates a consistent approach [20]. However, the definition of a caregiver includes a child of 16 years old who is the head of a CHH. Placing the responsibility to consent for their younger siblings to participate in health research on another child (the 16-year-old) is onerous; particularly if the study poses a slight increase over minimal risk. In such circumstances, it would be more appropriate that the child head of the household nominate a trusted adult to assist them as recommended in national Good Clinical Practice Guidelines for Clinical Trials [21].

Researchers may also apply to the REC for a parental waiver in favour of an adolescent self-consent strategy. Approval for this is contingent on the following conditions [6]:

(i) The nature of the study should be such that it is desirable and ethically justifiable to allow self-consent, for example, when research is ‘sensitive’ and it would be difficult to enroll adolescents with parental consent because of their unawareness of behaviour (such as substance abuse);
(ii) The participants are in most instances 16 and older;
(iii) The study (generally) poses no more than minimal risk; and
(iv) There is evidence of ‘prior engagement with participating community role players which indicates that ‘independent consent is acceptable to the parents’.

The CIOMS guidelines [16] permit adolescent self-consent in circumstances where parental consent is ‘undesirable’ and therefore self-consent would be appropriate. They offer examples, first, where local law considers children to have the legal status of adults, that is, emancipated minors. In this instance, as the law considers them to have full legal capacity to make all decisions on their own, they are able to consent to research without any assistance [16]. We disagree with the approach taken by the CIOMS guidelines regarding emancipated minors for several reasons. In some countries the standard for emancipation is very low, for example, in Kenya, where pregnancy emancipates a female minor [22]. This change in her legal status makes a vulnerable child an adult without any special legal protection. Additionally, while the concept of mature minors rests on an easement of capacity, the guidelines do not provide any assistance in interpreting this norm.

Waivers are also permitted when the nature of the research is such that parental awareness of the child’s belief or behaviour may place the child at risk of harm, for example, where the research focuses on illicit drug use, terminations of pregnancy or family violence [16]. Allowing a REC to approve a self-consent strategy with adolescents is important as it allows certain forms of research to be conducted which would otherwise not be feasible [2, 7, 8, 11–14]. This is particularly important in the context of South Africa’s inflexible legal framework [1–4, 7–14]. The approach taken in the guidelines is broad leaving RECs with considerable flexibility. The guidelines ask RECs to determine if the self-consent approach is both desirable and ethically justified. We suggest that the term desirable means that the self-consent approach is in the circumstances worthwhile or valuable. This could be both for participant’s well-being and for knowledge generation. RECs would need to rely on the norms in the national ethics guidelines and general ethical principles (autonomy, beneficence, non-maleficence and justice) to establish if the approach is ethical. This is in line with CIOMS guidelines [16].

South African guidelines also describe three protections which should be in place if a parental waiver is granted: the participants ought to be older adolescents; the risk level should preferably not exceed minimal risk; and researchers should demonstrate that there has been community engagement which shows support for the parental waiver [6]. These norms are also to a large extent consistent with the principles in CIOMS. However, the community consultation requirement is not a norm in other guidelines.

The breadth and flexibility of guidelines also means that there are a lack of established factors which could be used to answer questions, such as, would logistical challenges in obtaining parental consent be relevant justification to request adolescent self-consent to a minimal risk online study? The protections outlined in guidelines are also somewhat limited. The recommendation that adolescents be 16 or older for self-consent is concerning as there is no guidance on when a committee may go below this age. This means it is unclear whether a REC could approve research into the accessibility and acceptability of contraceptive services by 12–15 year olds who are lawfully obtaining this service in terms of the Children’s Act [17] but may be engaging in under-age sex in terms of the Sexual Offences Act [23], and therefore not be willing to participate if parental consent was necessary.

The guidelines [6] require strengthening in two other ways. First, although there are protections in place, unlike CIOMS [16], they do not? address the need to support adolescent decision-making. Research with adolescents in South Africa and the US found that ‘most adolescents indicated that if parental permission was not required, they would want to talk with someone else about the study, such as a trusted adult’ [24]. Based on these empirical findings, guidelines should include additional protections such as counselling or encouraging adolescents to discuss participation with a trusted adult [24]. Second, the requirement that researchers must provide evidence of community engagement is a norm unique to South Africa [25]. There are a number of challenges with this community consultation norm. It is difficult to operationalise because establishing appropriate community representatives if a community advisory board (CAB) does not exist can be complex [26] and guidelines on what constitutes appropriate evidence of consultation are limited. There is anecdotal evidence that RECs do not have a common understanding of how to apply this norm, resulting in inconsistent approaches.

## Discussion

This discussion is based on an assumption that parental or guardianship consent is generally the gold standard for research with children. The rationales for this protective approach are articulated in the CIOMS guidelines [16] which state that relative to adults, children are at increased risk to research-related harm and given their lack of full legal capacity, they may be less able to protect their own interests through the informed consent process. However, mandatory parental consent limits the nature of research which can take place with children [2, 7–9, 11–13, 15] and the ability to enroll and retain children in research [7, 8, 13, 15, 25, 26]. This confines advancements in child health including developing or improving health care services for children.

In establishing whether ethical norms on parental waivers are fit for purpose, we explore three sub-questions: (1) when are parental consent waivers appropriate? (2) what protections are needed if parental consent is waived? and (3) what procedural requirements should be met if a parental waiver is granted?

Our underlying approach is to (1) examine how the ethics guidelines and the flexibility they offer could be strengthened through the development of additional factors to guide decision-making, and (2) identify the broader issues which may be applicable to other countries wishing to evaluate their own ethical norms regarding parental waivers.

(i) When are parental consent waivers appropriate?

As stated above, the grounds for parental waivers in our national ethics guidelines [6] are largely in line with principles in international ethics guidelines [16]. The circumstances in which parental waivers in favour of caregivers are appropriate, are clearly articulated in the South African guidelines [6]. Using a definition of caregiver in the Children’s Act is a strength of guidelines as it ensures clarity on the qualifying persons for proxy parental consent. CIOMS requires a ‘legally authorised person’.

However, waivers in favour of adolescent self-consent are less clear. The literature has identified some useful factors relevant in establishing if adolescent self-consent is ethical, including an examination of both the study and the potential child cohort. In terms of the study, relevant factors for consideration include: the nature of the study and whether parental consent is desirable or feasible [27, 28]; whether a parental consent approach will compromise scientific validity [29]; the risks, benefits and the risk–benefit ratio of the study [27, 28]; and any proposed protective measures established to support adolescent decision-making [29]. With risks, a distinction should be made between the level of risk posed by the study and the risks, if any, posed by behaviours of participants [22, 29]. In terms of the adolescent participants*,* relevant factors include: the capacity, maturity and cognitive ability of the child participants including whether any of the participants will have full legal capacity in terms of local law [22, 29] and a review of the best interests of the potential child participants [29].

Bauman, Mellins, and Klitzman [29] suggest that parental waivers are ethically acceptable if there is an appropriate risk level, the adolescent has capacity and if obtaining parental consent is not in the best interests of the child *or* if parents are unable to provide permission. We contend that a key factor to consider is the capacity requirements for consenting to the study. Hunter and Pierscionek [30] argue that a child would have capacity if they could show that they understood (1) the nature of the research, (2) their rights as a participant and (3) the risks and benefits of participation. We argue that a waiver approach based on capacity is in line with the principles of a child’s evolving capacity and their right to participate in decisions that affect them [17, 31]. Still, Hunter and Pierscionek caution against individual capacity assessments being undertaken by researchers who they feel may be biased by their own self-interest in the outcome [31]. Hence they suggest that the assessment ought to be undertaken by a person independent to the trial [31].

(ii) What protections are needed if parental consent is waived?

Following the approach in CIOMS [16], we argue that protections be established if parental consent is waived. The protections need to be in place for waivers in favour of caregivers and for adolescent self-consent. CIOMS guidelines [16] provide two examples of possible protections in the form of decisional support for adolescents. These are the appointment of another adult (other than a parent) nominated by the child or the engagement of an *independent* psychological or medical practitioner to support participants [16]. Furthermore, the standard for assessing whether the protections are sufficient is whether they ensure child research participant’s best interests are protected [16]. When allowing proxy consent by adult parental substitutes, the required protection should be to ensure that the adult is legally or ethically authorised to provide the consent. In the South African context, this would mean that the child head of a CHH cannot consent for their younger siblings to participate in research, even if they are considered a caregiver in terms of the Children’s Act. When children are to self-consent, decisional supports should be required. These can take many forms, for example, peer support groups [32] or the support of another trusted adult.

(iii) What procedural requirements should be met if a parental waiver is granted?

As stated above, the CIOMS guidelines [16] do not require additional procedural protections. In the South African context, the procedural requirement of community consent seems to offer very little, if any, additional protection. This is largely because it is not implementable without more detail on who researchers should consult with to obtain this approval nor the rationale for obtaining community consent. The guidelines do not specify who would comprise the relevant community, and whether community consent would (a) be a substitute of parental consent or (b) provide permission for adolescents to self-consent? If the former, it is unclear which community representatives would be the most appropriate proxies for parental permission (e.g., would representatives need to themselves be parents?). In relation to identifying particular community representatives for consultation purposes, it is unclear how this would be operationalised for national research or when target populations are accessed virtually (e.g., adolescents using online platforms). The vagueness of the community consultation norm in guidance has led to inconsistency in its application. Although community engagement is critical to research, requiring it as a precursor to an adolescent self-consent approach appears arbitrary.

## Conclusions, lessons learned and recommendations

Within the context of South Africa’s restrictive legal framework, the norms on parental waivers in the national ethics guidelines are a much needed flexibility. However, despite their flexibility when compared to the legal norms, they lack detail which could assist in the ethical evaluation of such protocols. In terms of law reform, we strongly support previous recommendations that the National Health Act be amended to facilitate a more nuanced approach to the participation of children in health research [1–4]. We also recommend reforms to guidelines to ensure clarity and consistency, and that additional protections be mandated when an adolescent self-consent strategy is deemed most suitable**.**

Finally, we have identified lessons learned from our review of the South African ethical-legal framework. First, it is important for all ethical-legal frameworks to facilitate parental waivers in certain circumstances. Two, the principle of allowing a waiver of parental consent should be set out in law but the detail regarding the relevant ethical factors to be considered on a case-by-case basis should be described in ethics guidelines. The challenge in the South African context is that the law and ethics guidelines are not harmonized. Therefore, laws should not be overly restrictive. Three, researchers should carefully consider how to protect children’s best interests when a parental substitute is appointed or self-consent is permitted. These protections should be specified in ethics applications. Four, guidelines should set a range of factors to be considered in the assessment of whether the self-consent strategy is ethical. Five, procedural protections which require approval from the broader community where the research is being conducted should not be linked directly to the issues of parental waivers. Six, more research is required on the concept of the best interests of the child and how this would apply when evaluating research protections.

## Data Availability

Not applicable.

## Abbreviations

CHH: Child headed households
CIOMS: Council for International Organisations of Medical Sciences
OVCs: Orphans and vulnerable children
RECs: Research Ethics Committees
